# Identification of Candidate Genes Associated with Yak Body Size Using a Genome-Wide Association Study and Multiple Populations of Information

**DOI:** 10.3390/ani13091470

**Published:** 2023-04-26

**Authors:** Xinrui Liu, Mingxiu Wang, Jie Qin, Yaxin Liu, Zhixin Chai, Wei Peng, Yixi Kangzhu, Jincheng Zhong, Jiabo Wang

**Affiliations:** 1 Key Laboratory of Qinghai-Tibetan Plateau Animal Genetic Resource Reservation and Utilization, Ministry of Education and Sichuan Province, Southwest Minzu University, Chengdu 610041, China; xinrui-liu@outlook.com (X.L.);; 2 Qinghai Academy of Animal Science and Veterinary Science, Qinghai University, Xining 810016, China

**Keywords:** yak, genetic resource, genome-wide association study, body size trait, genetic characteristic, extended model

## Abstract

**Simple Summary:**

The yak is a unique livestock species living in the Qinghai–Tibet Plateau. Investigating the morphological differences among different breeds of yaks is of paramount importance. However, due to the lack of effective communication of yak genetic information under natural and artificial selection, the genetic diversity of regional yaks is not effectively utilized for new breedings, and it is difficult for the existing analysis models to analyze such complex multi-species populations. Therefore, we extended the application scope of the current statistical model to perform whole-genome association analysis on multiple yak breeds and identified four genes significantly associated with body height. The findings of this study are of great significance for the development and improvement of yak morphological traits, as well as the expansion of statistical models.

**Abstract:**

Yaks have evolved several breeds or genetic resources owing to their geographical and ecological environment, and investigating the genetic construction of body size among breeds is key for breeding. Here, a genome-wide association study (GWAS) was performed for five body size traits in 31 yak breeds and genetic resources. The information from clustering individuals according to their habitats was used for kinship grouping in the compressed mixed linear model (CMLM). We named this approach the pCMLM method. A total of 3,584,464 high-quality single nucleotide polymorphisms (SNPs) were obtained, and six markers were found to be significantly associated with height by pCMLM. Four candidate genes, including *FXYD6*, *SOHLH2*, *ADGRB2*, and *OSBPL6*, were identified. Our results show that when CMLM cannot identify optimal clustering groups, pCMLM can provide sufficient associated results based on population information. Moreover, this study provides basic information on the gene localization of quantitative traits of body size among yak breeds.

## 1. Introduction

Yak (*Bos grunniens*), a unique large livestock species of the Qinghai–Tibet Plateau and surrounding Hengduan Mountains, provide a basic resource for the livelihood of plateau farmers and herders [1]. Due to different geographical and climatic environments, ecological conditions, grassland types, feeding levels, breeding levels, and social and economic structures in the main producing areas, China has formed 12 yak breeds: Qinghai Plateau yaks, Gannan yaks, Tianzhu White yaks, Bazhou yaks, Zhongdian yaks, Jiulong yaks, Muli yaks, Maiwa yaks, Niangya yaks, Xizang Alpine yaks, Pali yaks, and Sibu yaks [2]. Tibet is one of the main yak-producing areas in China accounting for 30% of the total number. Yaks are distributed in 71 counties in Tibet, forming many local yak breeds and populations, including the Niangya yak, Pari yak, Sibu yak, Sangsang yak, Sangri yak, Baqing yak, Dingqing yak, Kangbu yak, Jiangda yak, Leiwuqi yak, and Gongbujiangda yak [3]. 

This rich genetic diversity reflects adaptation to the external environment and is a vital genetic resource for breeding new breeds or strains. However, due to the lack of active communication of yak genetic information during natural and artificial selection, obtaining differential genetic information of different yak breeds and genetic resources has become very important. The yak body size trait is the most critical genetic index. It is an essential reference index to determine meat production performance and one of the most direct breeding selection parameters [4]. Some yaks are more aggressive due to mixing with wild blood, and extracting relevant biological traits is more complicated. However, technology for extracting animal body size traits by image recognition has become more advanced [5,6]. At present, digital images of beef cattle acquired by a Microsoft Kinect device can be used to establish model equations for predicting body weight, carcass weight, and body fat content; this has facilitated rapid and easy body size trait determination [7].

With the development of high-throughput genotyping technology, opportunities have been provided for identifying new genetic variants associated with economic traits in cattle, where single nucleotide polymorphisms (SNPs) distributed throughout the genome have become the genetic markers of choice. Genome-wide association study (GWAS) is a common experimental approach to study SNP markers associated with various economic traits in animal production by linking phenotypic and genotypic data and using statistical models to investigate genetic variant loci causally associated with the target trait [8]. The GWAS approach has successfully revealed genetic determinants associated with disease susceptibility and resistance in humans [9], animals [10], and plants [11]. At the same time, the total genome data of yaks is 2.7 G, indicating genetic diversity among populations and the use of random mating within populations. This results in a large number of effective SNPs within the yak population. This will increase the computational burden. The compressed Mixed Linear Model (CMLM) was reported to improve the statistical power and computational speed of GWAS by clustering individuals into groups based on kinship among individuals [12]. When the likelihood values of the testing model in the CMLM are not able to identify the optimum clustering group, the individuals are grouped in a group with only one individual. This result has been proved by simulation results. However, previous population clustering [13] is widespread in animals, especially in yaks, and application of previous population structure is key for animal GWAS.

In this study, we performed GWAS using yak datasets from different geographical areas in order to (1) detect SNP markers associated with body size among yak breeds, and (2) develop animal GWAS methods using the information provided on the populations.

## 2. Materials and Methods

### 2.1. Individual Samples and Sequencing

A total of 94 yaks were collected from different regions of the Qinghai–Tibet Plateau in China, including 17 Tibetan regions, 4 Qinghai regions, 4 Sichuan regions, 2 Gansu regions, 2 Xinjiang regions, 1 Yunnan region, and 4 wild yaks; in total, there were 31 yak breeds and genetic resources (Table 1). Detailed information on these yak genetic resources and their distribution areas are shown in Table S1. Population samples were obtained from the Key Laboratory of Qinghai–Tibetan Plateau Animal Genetic Resource Reservation and Utilization, Sichuan Province, and Ministry of Education, Southwest Minzu University. The sequence files of 94 yaks [13] were all obtained from the sequencing results of DNA extracted from blood samples using the Illumina Hiseq 2000 sequencer (Illumina, San Diego, CA, USA). Individuals of each yak (more than 2.5 years old, male) breed or genetic resource were measured for body height (BH, cm), body length (BL, cm), body weight (BW, kg), chest circumference (CC, cm), and circumference of the cannon bone (CCB, cm). The phenotypic values used for GWAS analysis in this study were replaced by the overall mean values of the measured phenotypes for each breed or genetic resource. This allowed for a better representation of breed characteristics [14].

### 2.2. Genotyping Quality Control and Filtering

Data filtering was performed using the FASTP [15] software (version 0.20.1). Double-end sequencing reads were aligned using the Burrows–Wheeler Alignment Tool (BWA) [16] software (version 0.7.15), and high-quality reads were compared with the yak reference genome (BosGru3.0); the resulting BAM files were sorted using the sort command of the SAMtools [17] software (version 1.11) and de-duplicated using the rmdup command (all duplicate reads were removed directly). Local recombination of reads and comparison of near enhanced indel polymorphisms were performed using the Genome Analysis Toolkit (GATK) [18] software (version 4.0.1). Then the HaplotypeCaller command in GATK was used for SNP calling, CombineGVCFs command for VCF file merging, GenotypeGVCFs command for variant detection, and VariantFiltration command for initial filtering. The variant filtering conditions were set as follows: QD (QualByDepth, variant loci confidence divided by the number of unfiltered non-reference reads) < 2.0; FS (FisherStrand, Fisher exact test to assess the probability that the current variant is a strand bias, this value is between 0 and 60) > 60.0; MQ (RMSMappingQuality, square root of the matching quality in all samples) < 40.0; MQRankSum (MappingQualityRankSumTest, assesses the confidence based on the matching quality of the read of REF and ALT) < 12.5; ReadPosRankSum (ReadPosRankSumTest, evaluate the variation confidence by the position of the variation in the read, usually the error rate is higher at both ends of the read) < 8.0, and SOR (StrandOddsRati, comprehensive assessment of the likelihood of strand bias) > 3.0. Maker filtering using the PLINK [19] software (version 1.90) with the variant filtering conditions were set as follows: maf (minor allele frequency) 0.05, max-missing (maximum deletion rate of genotype) 0.05, and hwe (deviations from Hardy–Weinberg equilibrium) 1 × 10^−6^. The Genome Associated Prediction Integrated Tool (GAPIT) [20] was used for heterozygosity analysis of all markers.

### 2.3. Population Structure

The neighbor-joining (NJ) tree was constructed using the P distance matrix calculated by the VCF2Dis [21] software (version 1.46), tree beautification was performed on the online site iTol (https://itol.embl.de/, accessed on 3 October 2022), and principal component analysis (PCA) was performed and plotted using the GAPIT package in R. Population clustering analysis was performed using the Admixture [22] software (version 1.30). Kinship and differentiation between samples from different regions were viewed by joint analysis, and linkage disequilibrium (LD) decay analysis was performed using PopLDdecay (version 3.41) [23].

### 2.4. Association Study

Genome-wide association analysis was performed using a compressed mixed linear model (CMLM) [12] in the GAPIT (version 3.0) software, where PCA and the kinship matrix were added as covariates, *p*-values were corrected with Bonferroni, and the cutoff was set to 0.05/number of all markers. 

The general expressions of CMLM are consistent:

(1) $$Y=\mathrm{Wv}+{\mathrm{SNP}}_{i}+\mathrm{Zu}+e$$

where, Y is the phenotypic vector (n × 1); W is the covariate design matrix of vector v, and v is the corresponding coefficient vector, which is the non-marker effect among the unknown fixed effects (we used the effects of the first 3 PCs as fixed effects in this study); ${\mathrm{SNP}}_{i}$ is the testing marker genotype; Z is the random design matrix (n × n) of u, where we re-defined the Z matrix as n × n′, n′ being the number of groups compressed, and u being the random effect vector of individuals, which obeys u~N(0,K$V_{g}$), of which K is the n × n kinship genetic matrix (in this study, we used the group kinship matrix n × n′ to replace K); $V_{g}$ is the additive genetic variance, and e is the random residual and obeys e~N(0,I$V_{e}$), in which I is the n×n design matrix and $V_{e}$ is the residual component.

### 2.5. Population Index Building

Kinship in the model was calculated using all the markers. Combining test markers with kinship in MLM may lead to confusion between test markers and the genetic effects of individuals defined by kinship. In CMLM, Zhang et al. [12] introduced a variable now called the compress group, which clusters individuals with closer kinship into groups and uses the kinship between groups instead of the kinship between individuals for the operation. In this study, 94 individual yaks were artificially clustered into 7 populations based on their geographical relationships: Sichuan, Qinghai, Tibet, Gansu, Yunnan, Xinjiang, and wild yaks. We added a “compress_z” variable to the GAPIT built-in function GAPIT.Compress.R to store the real geographical groupings; this provides the groupings in CMLM in advance instead of the compressed groupings being estimated. We named this approach the provided compressed mixed linear model (pCMLM). The specific parameter compress_z under this method was marked with the number 1 to be identified as the same group of objects, and the output parameter group.membership was marked with the same number to be the same group. Currently, the parameter file for pCMLM has been uploaded to GitHub (https://github.com/liu-xinrui, accessed on 8 February 2023), which can provide a real or artificially defined grouping file for association study, as well as add grouping information to CMLM.

### 2.6. Identification of Candidate Genes

Based on the physical location of the target trait association loci on the yak reference genome and combined with the LD decay distance of the yak genome (~20 kb), associated genes were screened on both sides of the SNP loci; we adjusted the LD decay distance to 100 kb when no linked genes were detected. If the gene on the reference genome had only an Ensembl ID, the sequence of the gene was extracted for Basic Local Alignment Search Tool (BLAST) comparison using the wild yak reference genome (BosGru_v2.0) for functional analysis. For multiple significant SNP loci, haploid block mapping was performed using LDBlockShow [24] (version 1.40) for all SNPs within 100 kb upstream and downstream of the lead SNP, and a box plot of independent significant SNPs and phenotypic values of each individual within a block was created.

## 3. Results

### 3.1. Phenotypic Distribution

We analyzed five body size traits of 94 adult yaks and summarized the descriptive statistics (mean, variance, maximum, minimum, and coefficient of variation) for different body size traits (Table 2). Most records of the body heights ranged from 100 to 205 cm. The mean value of body height was 118.2 cm, the mean value of body length was 138.6 cm, the mean value of body weight was 284.9 kg, the mean value of the chest circumference was 168.8 cm, and the mean value of circumference of the cannon bone was 17.24 cm. All body size data of wild yaks were higher than those of domestic yaks in all regions. The t-test results showed that there was a significant difference (*p*-value < 0.05) between the body sizes of the domestic yaks and wild yaks. Among them, the body size trait of yaks in the Tibetan region was in the upper level among domestic yaks.

### 3.2. SNP Calling and Population Structure

**Figure 1:**
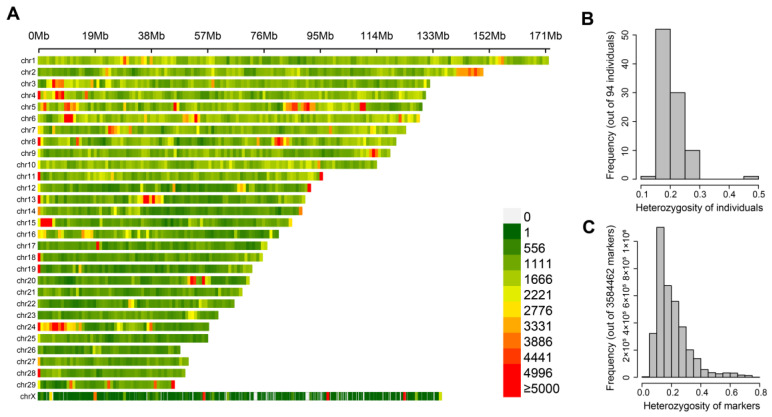
Distribution of genotypes in the whole genome and frequency of heterozygosity. (**A**) SNP marker density represented by relative physical position on chromosomes. The more the color leans towards red, the higher the density of markers in the window. Each color block indicates the number of SNPs within a 1 Mb windows size. (**B**) Histograms of heterozygosity frequencies for all 94 individuals. (**C**) Histograms of heterozygous frequencies of all SNP markers.

A total of 47.15 million markers, including SNPs, indels, and other variants, were detected using the BWA-SAMtools-GATK pipeline [25] program with default parameters. Overall, 3,584,464 SNPs remained after filtering by the GATK and PLINK software; on average, they were distributed over 29 autosomes and 1 X chromosome, and the SNP density of most windows was >1 kb/Mb (Figure 1A). In addition, the heterozygosity of most individuals’ and SNP markers’ was low (Figure 1B,C).

**Figure 2:**
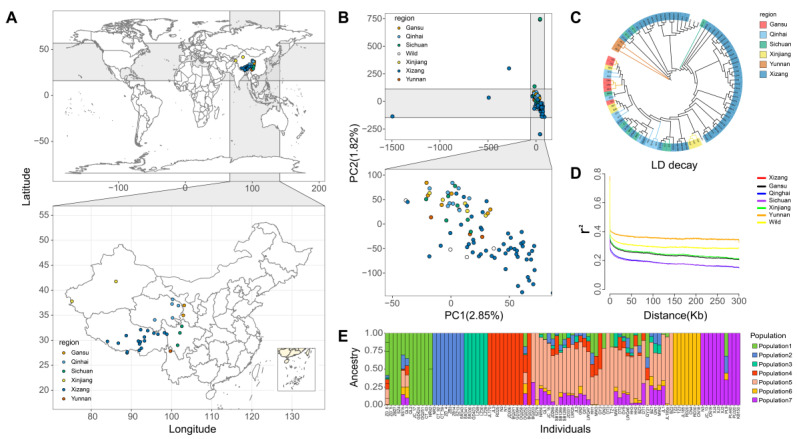
The location distribution of sample collection and population stratification. The distribution map of the breeds was drawn using the latitude and longitude of the sampling sites (**A**). Population structure explained by PCA using all SNP markers (**B**). All SNP markers were used to generate NJ-tree of 94 yaks using VCF2Dis software (**C**). All SNP markers were used for LD decay analysis of cultivars from seven different provinces and regions (**D**). All SNP markers were used to cluster the cultivars from seven different provinces by Admixture software. Different colors are used to indicate different provinces and regions in the figure (**E**).

In this study, yak populations were derived from 31 yak breeds and genetic resources from different regions of the Qinghai–Tibetan Plateau, and consisted of several yak populations from several provincial areas of China (Figure 2A); moreover, this sample had complex population structures. To analyze the population structures of the 94 yaks, we performed PCA, population stratification, and NJ-tree analysis on seven yak populations (including six regions and wild yak populations) using 3.58 million high-quality SNP data points obtained through filtering. The two-dimensional scatter plot of PCA clustering showed that the population structure of the yak populations was relatively weak, and it was difficult to distinguish between population structures. After excluding outliers, the six populations were in a mixed state excepted for Tibetan individuals that could be roughly clustered into a population (Figure 2B). The genetic variance contribution explained by the first two principal components was 2.85% and 1.82% (Figure S1). The NJ-tree clustering results showed that most of the breeds or genetic resources shared a recent common ancestor with both the Tibetan and Gansu populations, and all individuals were not independent; these NJ-tree clustering results presented approximately the same population structure as PCA did (Figure 2C). The optimal CV value of 1 in admixture population structure analysis cannot accurately reflect the actual grouping of the population. To analyze yak breeds and genetic resources more accurately, we forced grouping of Admixture, and classified yak breeds and resources based on geographical population (seven populations in total). However, the results only showed that most Tibetan individuals could be clustered into one population, while the remaining six populations were in a mixed state where specific clustering situations could not distinguish (Figure 2E). The LD analysis showed that the breed-based yak populations had more rapid decay of LD and lower LD levels. The most immediate decay was in the Tibetan breeds or genetic resources, followed by the Sichuan yaks, Qinghai yaks, Gansu yaks, Xinjiang yaks, Wild yaks, and Yunnan yaks.

### 3.3. GWAS and Candidate Genes

**Figure 3:**
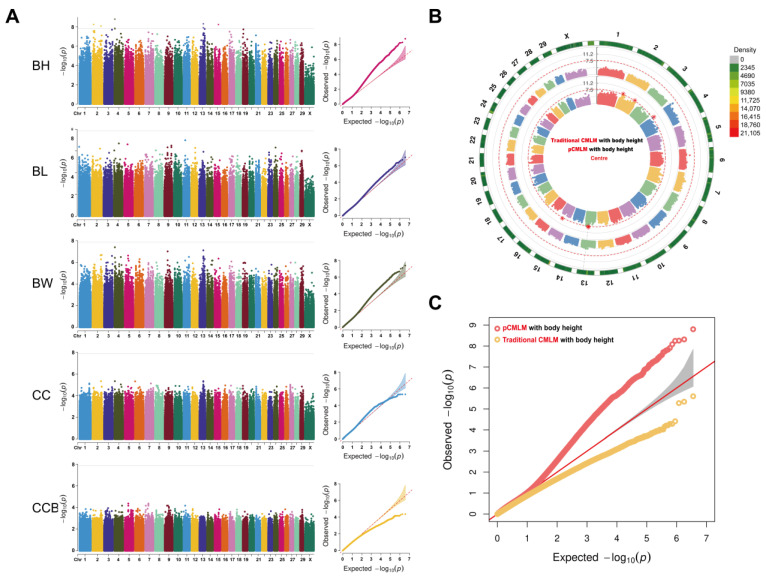
Manhattan and quantile-quantile plots of the *p*-values for the genome-wide association study of BH, BL, BW, CC, and CCB of yaks based on the pCMLM method; the horizontal line of significance threshold (*p*-value < 1.39 × 10^−8^) was used to distinguish significantly associated loci, and the different colors to distinguish different chromosomes (**A**); Circular Manhattan (**B**), and quantile–quantile plots (**C**) of the optimized pCMLM method and the conventional CMLM method in detecting body height traits in yaks, where the inner ring is pCMLM, the outer ring is conventional CMLM, and the outermost ring indicates the labeling density of this chromosome.

The CMLM model was used to associate loci with body size traits, and two statistical strategies were constructed. The first strategy was a tight grouping constructed by splitting all individual kinship matrices into several inter-group kinship matrices using CMLM, named the CMLM group. The second was CMLM using a prior population clustering parameter, named the pCMLM group. Comparing the negative twice likelihood (-2LL) of the two strategies for detecting BH traits, the results showed that the difference between pCMLM and CMLM was less than 10% of the mean value. Genotypic and phenotypic data of 94 individual yaks were analyzed using the GAPIT software with the first three principal components as fixed effects. Manhattan and quantile–quantile (QQ) plots are shown in Figure 3. After Bonferroni correction, six SNPs were found to pass the 5% threshold line (*p*-value < 1.39 × 10^−8^) and these were associated with BH only in pCMLM (Figure 3A), whereas no significant loci associated with body sizes were detected in any of the CMLM (Figures S2–S6). The QQ plot (Figure 3C) of pCMLM shows that all points corresponding to observed and predicted values were in the middle or above the diagonal line. Although there are some deviations, the model still has a meaningful corrective effect on population stratification relative to traditional CMLM. The likelihood value used to determine the best compression ratio in traditional CMLM was not significant. CMLM does not catch the best clustered groups and cannot reflect the true population structure; this could lead to a large number of false negative results (Figure 3B). The QQ plot of traditional CMLM with BH show most points below the diagonal (Figure 3C), which indicates that the observed *p*-value for most loci was less than the expected value and that the model overcorrected for this group. The significant SNPs detected in pCMLM were rs769892 (located at 4,883,046 bp) on chromosome 4, rs2659279 (located at 59,118,279 bp) and rs2659285 (located at 59,119,427 bp) on chromosome 13, rs310769 (located at 16,165,590 bp) and rs477265 (located at 57,505,237 bp) on chromosome 2, and rs2910497 (located at 129,838,648 bp) on chromosome 15. The significant SNPs with their associated candidate genes did not have annotation information in the 100 kb linkage disequilibrium interval information either upstream or downstream. Therefore, we extracted the FASTA sequences of these candidate genes that were not annotated with gene names on the reference genome, for sequence alignment with the reference genome of wild yaks using BLAST. Except for the rs769892 locus, all the candidate loci obtained gene names with high similarity on the wild yak reference genome (Table 3).

### 3.4. Genotype Correlation in the LD Block

**Figure 4:**
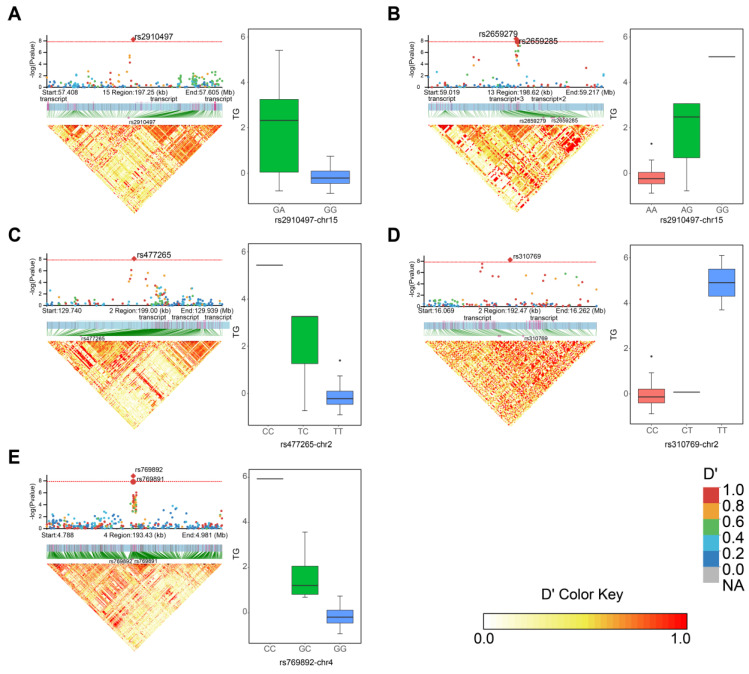
Local Manhattan plot of 200 kb near each independently significant SNP (top left), the structure of the transcript in this region (middle left), and the LD heat map (bottom left). Phenotype distribution among the genotypes of all independently significant SNPs is on the right side. The red horizontal line indicates the threshold (*p*-value < 1.39 × 10^-8^) of GWAS. Multiple transcripts are abbreviated in the figure. The LD heat map distinguishes the strength of association with different colors: the redder the color, the stronger the association. The genotype-phenotype association plots of SNPs distinguish different genotypes in pink, green, and blue. rs2910497 of chromosome 15 (**A**), rs2659279 of chromosome 13 (**B**), rs477265 of chromosome 2 (**C**), rs310769 of chromosome 2 (**D**), and rs769892 of chromosome 4 (**E**). The horizontal coordinate of the box line plots indicates the allele distribution of the marker on all individuals, and its vertical coordinate indicates the phenotypic values of all individuals of the marker.

In order to further determine the haplotype effect of association markers in BH, we used phenotype values distribution of significant alleles across all individuals to show the influence between BH and alleles. The results of haplotype analysis of 100 kb before and after the independently significant SNP showed that there was linkage disequilibrium in chr15-rs2910497-57505237 (chr: chromosome of the SNP; rs: SNP numbering on the genome; the third digit indicates the relative physical position of the chromosome on which it is located). The nearby LD block region was small, and the SNP was located on the outer side of the transcript. The analysis of allele haplotype based on this SNP showed that only GA and GG genotypes existed in 94 individuals, and no homozygous AA genotype was observed. In the results of the BH traits, yak individuals show higher body height (Figure 4A) when they have allele A at this SNP position. The same situation was also observed for four SNPs: rs2659279 (Figure 4B), rs477625 (Figure 4C), rs310769 (Figure 4D), and rs769892 (Figure 4E); the four haplotypic alleles with strong additive effects on BH were G, C, T, and C, respectively. There was a strong LD between chr13-rs2659279-59118279 and chr4-rs769892-4883046, two SNPs in a LD block, but the block segment was small. Among them, rs2659279 and rs2659285 on chromosome 13 had strong linkage disequilibrium, and the two SNPs were physically close to each other. rs2659279 was used as a benchmark when screening candidate genes. Except for rs769892, the other four SNPs with statistical significance were located near the intron region where at least one unknown transcript existed, and the sequences of the physically closest transcripts were compared by blast. The results showed that the transcript near rs2659279 was matched to *FXYD* gene, rs310769 to *ADGRB2*, rs2910497 to *SOHLH2*, and rs477265 to *OSBPL6* (Table 3).

## 4. Discussion

Most GWAS models are effective in detecting populations, which is usually conducted by using genome-wide SNPs in a large number of individuals in the same population [26]. They have good statistical power in a wide range of applications for locating human disease loci [27] and developing molecular markers [28], and for breeding selection of major economic plants and animals [29]. On the Qinghai–Tibet Plateau, it is difficult to obtain a large number of individual yaks of the same breeds or genetic resources. In this study, we performed GWAS with a large number of non-homologous resource individuals. From the results, even if there is significant population stratification, it is difficult to reduce the stratification effect by marker estimation through traditional GWAS models. However, population structure is often a fundamental factor affecting the accuracy of association results, which inevitably leads to false positive and false negative association results [30]. In addition, all of these yak populations were sampled from each kernel population. There is wide inbreeding in each kernel population to keep pure lines between families. All samples were clustered into several groups. Hence, heterozygosity is not abundant.

Variation in body size traits is mainly based on distant cross-breeding among yak populations or genetic resources. In particular, to rejuvenate current domestic yak production performance, the wild yaks with superior traits were used for cross-breeding by local herders. These wild-blooded yaks (cross-breeding offspring of wild yaks and domestic yaks) tend to have a stronger body. Therefore, yak populations are more complex throughout the whole Qinghai–Tibet Plateau region. None of the 31 yak breeds and genetic resources in this study showed significant population clustering in multiple cluster analyses, with only the Tibetan breeds and genetics resources having a more similar genetic structure owing to their domesticated origins in Tibet [13]. Such populations with complex population structures are not conducive to statistical analysis by traditional CMLM. The grouping process of CMLM, which assigns individuals with similar characteristics to the same group, uses the elements in the kinship matrix as a similarity measure [31], replacing the kinship between individuals [12]. The genetic principle utilizes intra-group balance, which reduces the variance of the model’s residual part and improves the statistical power of GWAS. The difference between pCMLM and CMLM is that pCMLM provides clustering relationships of breeds or populations from previous and known study, and the kinship is compressed directly. In contrast, CMLM needs to filter the best compression levels by optimizing the likelihood values. However, sometimes the likelihood values between different compression levels are insignificant. The above situation would cause CMLM to use individuals to represent groups and kinships without any compression, taking CMLM back to MLM. Population structure or population origin in yak populations usually implies similarity in nutritional level and growth environment [32], and so using these factors to force CMLM to be compressed is beneficial for detecting candidate genetic markers. However, the complex structure makes the statistical power of CMLM closer to that of standard MLM. Therefore, when traditional CMLM cannot identify optimal clustered groups, our proposed pCMLM can reduce the variance of the residuals in the model by providing the real kinship and group structure. In summary, pCMLM can provide us with more adequate association results. The results show a small difference in -2LL between the two strategies. The -2LL is used to determine how well the model fits the variables, and when there is a large difference in the -2LL values, the model’s fit can be used to explain the training optimality of the model itself. However, this training optimality is not absolute because the training optimality of the model itself does not fully represent the detection ability and prediction ability. In 2010 and 2014, Zhiwu Zhang and Meng Li, respectively, confirmed a negative correlation between -2LL values and GWAS detection efficiency using a large amount of simulated data, but this relationship was derived from a large number of statistics and no specific conclusion was given in a single experiment [12].

GWAS results based on pCMLM identified six SNP loci that were statistically significant in association with BH. Based on the annotation information provided by the 100 kb upstream and downstream of the yak reference genome, we obtained relevant annotation information on only four SNPs, but these contained only Ensembl ID. Probably because the yak reference genome is not yet complete, the sequences of these genes were compared to the wild yak reference genome by the BLAST tool. These candidate genes show association of rs2659279 (chr13-59118279) with *FXYD6* (domain containing ion transport regulator 6), rs2910497 (chr15-57505237) with *SOHLH2* (spermatogenesis- and oogenesis-specific basic helix-loop-helix 2), rs310769 (chr2-16165590) with *ADGRB2* (adhesion G protein-coupled receptor B2), and rs477265 (chr2-129838648) with *OSBPL6* (adhesion G protein-coupled receptor B2). Among these genes, the *FXYD6* gene is a member of the *FXYD* family encoding a transmembrane protein, a specific protein encoding the hippocampus phosphate that has been shown in humans to be involved in mediating the Na/K ion pump [33]. The *FXYD6* gene significantly accelerates Na^+^ deactivation and Na^+^ pump conversion rates [34] and alters the selectivity of the intracellular ion pump [35]. The *SOHLH2* gene belongs to the b-HLH (basic helix-loop-helix transcription factor) family, which encodes a testis-specific transcription factor essential for spermatogenesis, oogenesis, and folliculogenesis [36]. The b-HLH family is involved in numerous biological processes in the organism, including cell differentiation, cell cycle arrest, and apoptosis [37]. The *SOHLH2* gene has also been shown to play an important regulatory role in the reproductive gonadal axis, pituitary, hypothalamus, ovary, and testis of buffalo [38], pigs [39], and mice [36,40,41]. The *ADGRB2* gene acts as a transcriptional repressor through GA-binding protein, regulates vascular endothelial growth factor, and is significantly associated with its growth traits in grouper [42]. The *OSBPL6* gene is a member of the family encoding hydroxysteroid-binding protein (OSBP), an intracellular lipid receptor [43]. The *OSBPL6* gene contributes to the maintenance of cholesterol homeostasis in vivo by regulating cholesterol transport in humans through miR-33 and miR-27b [44]. In studies of *OSBPL6* in juvenile DePaul dwarf horses, it was shown that *OSBPL6* is an essential factor affecting body height in DePaul dwarf horses [45] by showing variable splicing of ES type in the pituitary gland. Moreover, ES variable splicing causes GH1 third exon jumping resulting in a 17.5 kD GH isoform, which is an essential factor contributing to height defects in patients with autosomal dominant growth hormone deficiency (type II) [46], and the gene may be associated with multiple epiphyseal dysplasias [47]. Therefore, it is hypothesized that *OSBPL6* and *ADGRB2* genes are the most likely candidates to affect body height traits in yaks. However, whether and how the studied localized genes affect body height and size traits in yaks needs to be further explored, providing new directions and ideas for later validation studies.

## 5. Conclusions

In conclusion, when traditional CMLM cannot provide effective compressed grouping by obtaining the best likelihood value, pCMLM can obtain better results by using the previous population clustering information provided. From the GWAS results of pCMLM and LD analysis, four candidate genes (*FXYD6*, *SOHLH2*, *ADGRB2*, and *OSBPL6*) were provided in association with yak’s body height. This study will help us to develop better biostatistical model optimization ideas and a deeper understanding of the relationship between genes and body height. These results may provide basic information for quantitative trait gene localization or candidate gene cloning in the yak body height formation mechanism.

## Figures and Tables

**Table 1:** Data distribution of yak samples.

Location ^1^	Breeds or Genetic Resources ^2^	Count ^3^
Tibet, China	Zhongba, Senza, Cuona, Sangsang, Sangri, Sibu, Riduo, Pali, Nierong, Longzi, Leiwuqi, Kangbu, Lijia, Jiangda, Gongbujiangda, Baqing, Dingqing	51
Qinghai, China	Qilian, Huanhu, Gaoyuan, Datong	12
Sichuan, China	Maiwa, Jiulong, Jinchuan, Changtai	12
Gansu, China	Tianzhu, Gannan	6
Xinjiang, China	Xinjiang, Bazhou	6
Yunnan, China	Zhongdian	3
Qinghai–Tibet Plateau	Wild yak	4
Total		94

^1^ The administrative divisions all represent the regional divisions of the provinces or are autonomous of the People’s Republic of China; ^2^ The names of the strains within the breeds or genetic resources are named after their geographical locations; ^3^ The number in count is the number of individuals contained in this study population in the region.

**Table 2:** Descriptive statistics of body size traits in yak.

Taxa *	Max	Min	Mean	SD	SE	CV (%)
BH	205	100.7	118.2	16.7559	1.72823	14.1805
BL	240	105.4	138.6	27.3521	2.82116	19.7283
BW	821	156.1	284.9	103.982	10.7249	36.4992
CC	270	140.1	168.8	23.2648	2.39958	13.7847
CCB	22.9	10.05	17.24	2.90050	2.39958	16.8203

* Body height (BH, cm), body length (BL, cm), body weight (BW, kg), chest circumference (CC, cm), circumference of the cannon bone (CCB, cm), maximum (Max), minimum (Min), standard deviation (SD), standard error (SE), coefficient of variation (CV). The description statistics of all phenotypes are generated by the summary function of R.

**Table 3:** Significant SNP and candidate gene information.

SNP No. *	Chr	Position (bp)	Alleles	Gene ID	Blast Gene Name
rs769892	4	4,883,046	G/C	-	-
rs2659279	13	59,118,279	A/G	ENSBGRP00000037978 ENSBGRP00000037933	*FXYD*
rs310769	2	16,165,590	C/T	ENSBGRP00000016273 ENSBGRP00000016168 ENSBGRP00000016219	*ADGRB2*
rs2910497	15	57,505,237	G/A	ENSBGRP00000032309 ENSBGRP00000032370	*SOHLH2*
rs477265	2	129,838,648	T/C	ENSBGRP00000000742	*OSBPL6*

* SNP No. indicates the sequence number in the entire tag list. Chromosomes and locations refer to physical location information in genomic data. The gene names are annotated from the GTF file of the Bosgru_v3.0 reference genome.

## Data Availability

There are 94 yak Bioprojects accessible at NCBI Bioproject (http://www.ncbi.nlm.nih.gov/bioproject, accessed on 1 March 2022) under accession numbers of PRJNA670822.

## Supplementary Materials

The following supporting information can be downloaded at: https://www.mdpi.com/article/10.3390/ani13091470/s1, Figure S1: Genetic variance explained and percentage of overall with Principal components of each dimension; Figure S2: Manhattan and quantile–quantile plots of body height of yaks by CMLM method; Figure S3: Manhattan and quantile–quantile plots of body length of yaks by CMLM method; Figure S4: Manhattan and quantile–quantile plots of body weight of yaks by CMLM method; Figure S5: Manhattan and quantile–quantile plots of chest circumference of yaks by CMLM method; Figure S6: Manhattan and quantile–quantile plots of circumference of cannon bone of yaks by CMLM method; Table S1: Distribution area of yak of different breeds and genetic resources.
