# Supplementary material for: Identification of Candidate Genes Associated with Yak Body Size Using a Genome-Wide Association Study and Multiple Populations of Information
Source: Animals (Basel). 2023 Apr 26;13(9):1470. doi: 10.3390/ani13091470 (PMC10177615; doi:10.3390/ani13091470)
Supplement: Supplementary file 1 [file animals-13-01470-s001.zip › animals-2297503-Supplementary.pdf]

Supplementary Material

Supplementary Table S1 Distribution area of yak of different breeds and genetic resources

Variety resources	Location (p-rovinces or autonomous)	Sampling area	Altitude (m)	Average annual team. (°C)
Zhongba	Tibet	Zhongba county, Shigatse, Tibet autonomous region	5000	-4 to 12
Shenza		Located in central Tibet of Shenza county, which between the Gangdis Mountains and the second largest lake in northern Tibet	4700	0.5
Cona		Located in the southern tip of the Tibet Autonomous Region and the Himalayas southeast of the Cona county	4380	-4 to 8
Sangsang		Ngamring County, Shigatse, Tibet autonomous region	4400 to 4600	6.5
Sangri		Sangri County, Lhoka, Tibet autonomous region is located at the southern foot of the Gangdis Mountains and in the middle valley of the Yarlung Tsangpo River.	>4065	8
Sibu		Mozhugongka County, Lhasa City, Tibet Autonomous Region is located in the middle and upper reaches of Lhasa River and the west side of Mila Mountain	3835	2 to 17
Riduo		Riduo Township, Mozhugongka County, Lhasa City, Tibet Autonomous Region	4370	0.8
Pali		Yadong County, Shigatse, Tibet Autonomous Region	3500	0
Nierong		Nyainrong County, Naqu, Tibet Autonomous Region	4700	-7 to 7
Longzi		Lhünzê County, Lhoka, Tibet autonomous region	3900	5.5
Leiwuqi		Riwoqê County, Qamdo, Tibet Autonomous is located in a branch of the Nyainqêntanglha Shanmai and the west of the Boshula Ridge	4500	2.5
Kangbu		Kangbu Township, Yadong County, Shigatse, Tibet Autonomous Region	>3500	-
Lijia		Lhari County, Naqu, Tibet Autonomous Region	4500	-6 to 8
Jiangda		Jomda County, Qamdo, Tibet Autonomous Region	3650	4.5
Gongbujiangda		Gongbo'gyamda County, Nyingchi, Tibet Autonomous Region	>3600	3 to 16
Baqing		Baqing County is located in the northeast of Tibet Autonomous Region, the east of Naqu District, and the upper reaches of salween	>4500	-1
Dingqing		Dênqên County, Qamdo, Tibet Autonomous Region	3850	3.4
Qilian	Qinghai	Qilian County is located in the north of Haibei Tibetan Autonomous Prefecture of Qinghai Province, bordering Gansu Province in the east and north	>2800	1
Huanhu		Gangcha County, Haibei Tibetan Autonomous Prefecture of	3300	-0.6

		Qinghai Province, is located in the north of Qinghai Lake		
Gaoyuan		Tibetan Autonomous Prefecture of Golog, Qinghai Province	4000 to 5000	-4
Datong		Datong Hui and Tu Autonomous County, Xining, Qinghai Province	2280 to 4622	4.9
Maiwa	Sichuan	Hongyuan Country, Tibetan Qiang Autonomous Prefecture of Ngawa, Sichuan Province	3500	2.9
Jiulong		Jiulong Country, Tibetan Qiang Autonomous Prefecture of Ngawa, Sichuan Province	1440 to 6000	4.9
Changtai		Baiyu County, Tibetan Qiang Autonomous Prefecture of Ngawa, Sichuan Province	3500	12.3
Jinchuan		Jinchuan Country, Tibetan Qiang Autonomous Prefecture of Ngawa, Sichuan Province	1950 to 5000	12.4
Tianzhu	Gansu	Tianzhu Zangzu Autonomous County, Wuwei, Gansu Province	2040 to 4874	3.5
Gannan		Maqu County is located at the east end of the Qinghai Tibet Plateau and the southwest of Gannan Tibetan Autonomous Prefecture	3700	1.2
Xinjiang	Xinjiang	Tajik Autonomous County of Taxkorgan, Kashgar Prefecture, Xinjiang Autonomous Rgion	3600	3.3
Bazhou		Korla City, Bayingol Mongolian Autonomous Prefecture, Xinjiang Autonomous Rgion, is Located in the middle of Xinjiang, the southern foot of Tianshan Mountains, and the northeast edge of Tarim Basin	934	11.4
Zhongdian	Yunnan	Zhongdian county in Diqin Tibetan autonomous prefecture	3450	5.5
Wild yak	Qinghai-Tibet Plateau	-	-	-

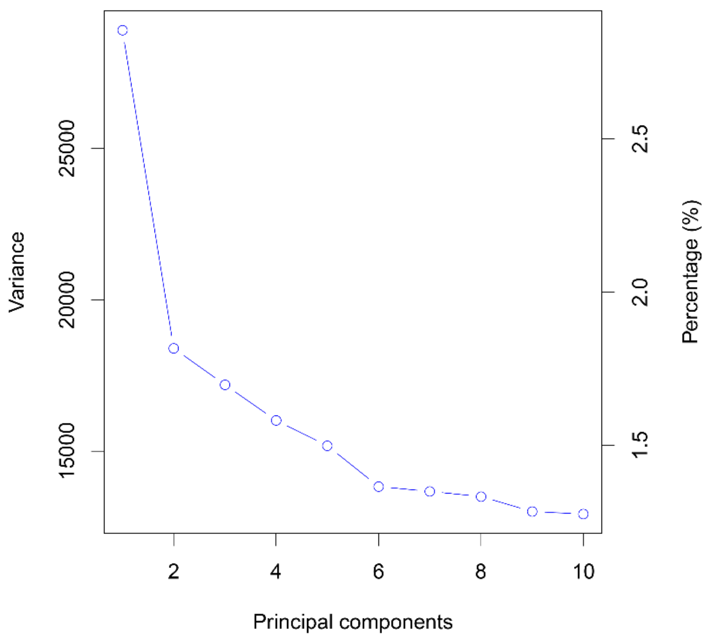

Supplementary Figure S1 Genetic variance explained and percentage of overall with Principal components of each dimension. The horizontal coordinates indicate the principal components of the different dimensions; The left vertical coordinate indicates the genetic variance explained by each principal component; The right vertical coordinate indicates the percentage of the overall variance accounted for by each principal component.

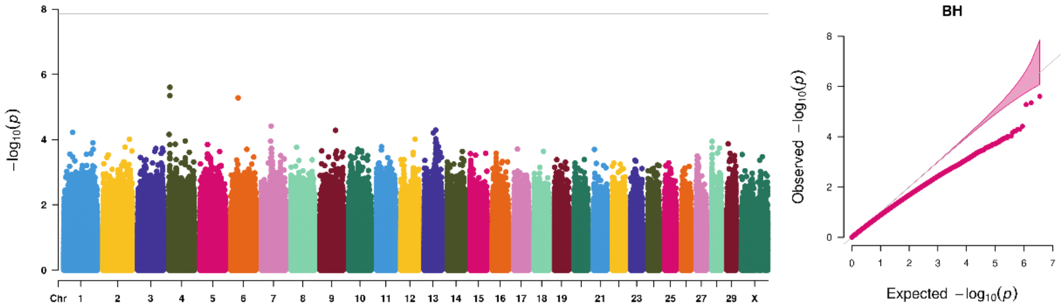

Supplementary Figure S2 Manhattan (left) and quantile-quantile plots (right) of the *p-values* for the genome-wide association study for body height (BH) of yaks based on the traditional CMLM method, the horizontal line of significance threshold (*p-value* < 1.39 × 10^{-8}) was used to distinguish significantly associated loci, and the different colors to distinguish different chromosomes.

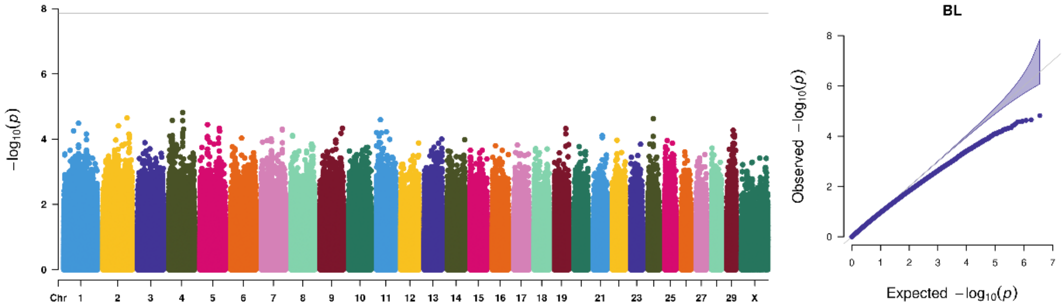

Supplementary Figure S3 Manhattan (left) and quantile-quantile plots (right) of the *p-values* for the genome-wide association study for body length (BL) of yaks based on the traditional CMLM method, the horizontal line of significance threshold (*p-value* < 1.39 × 10^{-8}) was used to distinguish significantly associated loci, and the different colors to distinguish different chromosomes.

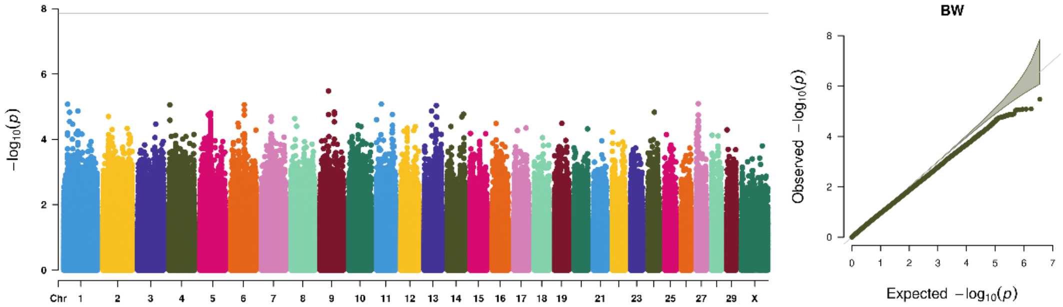

Supplementary Figure S4 Manhattan (left) and quantile-quantile plots (right) of the *p-values* for the genome-wide association study for body weight (BW) of yaks based on the traditional CMLM method, the horizontal line of significance threshold (*p-value* < 1.39 × 10^{-8}) was used to distinguish significantly associated loci, and the different colors to distinguish different chromosomes.

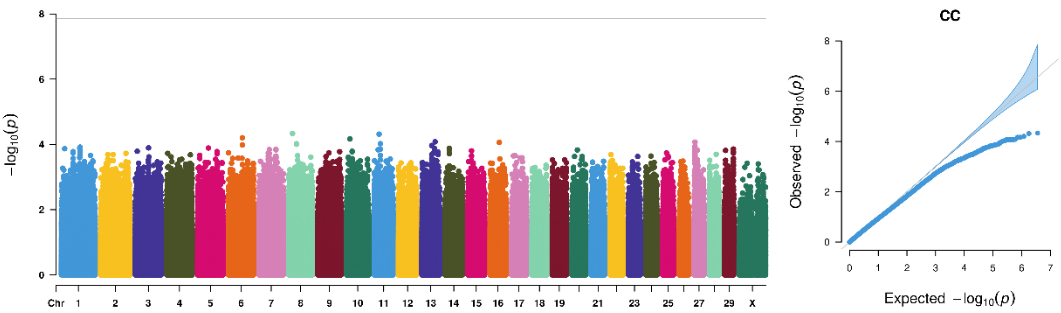

Supplementary Figure S5 Manhattan (left) and quantile-quantile plots (right) of the *p-values* for the genome-wide association study for chest circumference (CC) of yaks based on the traditional CMLM method, the horizontal line of significance threshold (*p-value* < 1.39×10^{-8}) was used to distinguish significantly associated loci, and the different colors to distinguish different chromosomes.

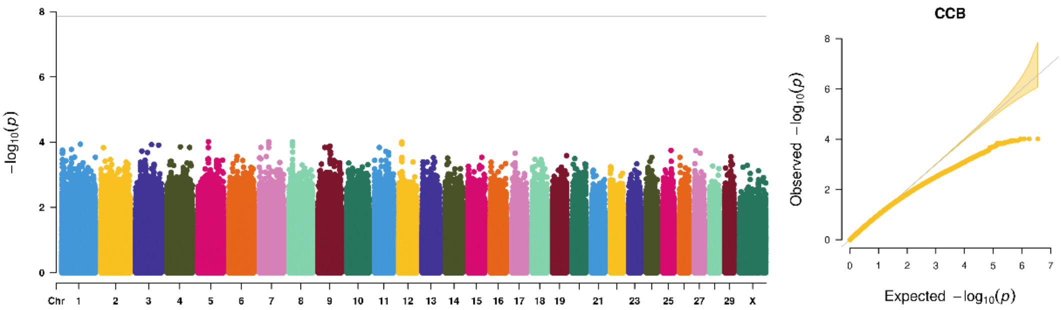

Supplementary Figure S6 Manhattan (left) and quantile-quantile plots (right) of the *p-values* for the genome-wide association study for circumference of cannon bone (CCB) of yaks based on the traditional CMLM method, the horizontal line of significance threshold (*p-value* < 1.39×10^{-8}) was used to distinguish significantly associated loci, and the different colors to distinguish different chromosomes.
